# Investigation on the fluorescence detection mechanism of NIR fluorescent probes based on intramolecular spiro cyclization

**DOI:** 10.3389/fchem.2025.1756681

**Published:** 2026-01-08

**Authors:** Zong-Wei Zhang, Yue Deng, Yong-Jin Peng, Yu-Ling Liu

**Affiliations:** 1 College of Intelligent Medicine, Jinzhou Medical University, Jinzhou, China; 2 Liaoning Province Key Laboratory of Human Phenome Research, Jinzhou Medical University, Jinzhou, China

**Keywords:** electronic structure, fluorescence, NIR fluorescent probes, quantum chemical calculations, ring opening/closing

## Abstract

**Introduction:**

This study focuses on the detection mechanisms of recently developed NIR fluorescent probes that depend on ring formation and opening processes. A novel class of polymethine dyes (NIRII-RTs) serves as the core fluorescent moiety of these probes, which exhibit bright, stable, and anti-solvent quenching NIR-II emission, accompanied by large Stokes shifts.

**Methods:**

Quantum chemical calculation methods were employed to systematically analyze the light absorption and emission processes of three target-specific probes: NIR-pH (targeting H^+^), NIR-ATP (targeting ATP), and NIR-Hg (targeting Hg^2+^).

**Results:**

The results demonstrated that the probes exhibit weak fluorescence in the closed spiro cyclization state. This weak emission is attributed to the interrupted π-electron distribution at the C-N bond of the reaction site, which facilitates electron transfer from the ground state to the excited state and restricts excitation to the benzene ring region. Upon reaction with target analytes, the spiro cyclization structure is disrupted, transitioning to a linear chain configuration.

**Discussion:**

The consistency between the calculated optical parameters and experimental data validates the proposed detection mechanism centered on spiro cyclization/ring-opening processes and associated changes in π-electron conjugation. This mechanism clarifies how the structural flexibility of the probes (driven by analyte binding) regulates their fluorescence properties, providing a theoretical basis for the rational design of high-performance NIR-II fluorescent probes with tunable optical responses. Future work may leverage this mechanism to develop probes for a broader range of analytes, further advancing their utility in biological imaging and environmental monitoring.

## Introduction

Fluorescent imaging has emerged as a cornerstone technology in modern life sciences and clinical medicine, enabling non-invasive visualization of biological processes, early disease diagnosis, and real-time intraoperative guidance (Nandanwar et al., 2026; Shi et al., 2026; Xie et al., 2026). However, traditional fluorescent probes operating in the visible (400–700 nm) spectral regions face inherent limitations that hinder their performance in complex biological systems. Visible light probes suffer from severe scattering and absorption by biological components (e.g., hemoglobin, melanin) and intense autofluorescence from tissues, leading to poor signal-to-noise ratios (SNR) and limited penetration depth (typically <1 cm) for applications such as deep tumor detection or vascular network mapping (Cheng et al., 2026; Li et al., 2026; Zhang Z. Y. et al., 2025; Zhao et al., 2025; Zheng et al., 2025).

The advent of near-infrared fluorescent probes has revolutionized biological imaging by addressing these bottlenecks. This spectral window coincides with the “optical transparency window” of biological tissues, where absorption by hemoglobin and water reaches a minimum, and spontaneous tissue autofluorescence is nearly eliminated. These unique properties translate to transformative advantages: NIR probes achieve penetration depths of 1–3 cm and spatial resolution down to 25–150 μm, enabling clear visualization of small blood vessels or tumor margins. (Zhang Y. T. et al., 2025; Zhang Y. H. et al., 2025; Zhang T. et al., 2025; Zhang S. S. et al., 2025; Zhang M. R. et al., 2025). Additionally, their longer wavelengths carry lower photon energy, minimizing phototoxicity and making them ideal for long-term *in vivo* dynamic monitoring, such as tracking immunotherapeutic responses. Clinically, NIR imaging has already demonstrated superior performance—for example, in glioma resections, NIR-guided surgery achieved 100% complete tumor removal, compared to 50% with traditional white light imaging.

Despite these breakthroughs, the development of activatable NIR probes—which switch from a “dark” to “bright” state upon binding disease-related analytes (e.g., pH, ATP, heavy metals)—remains challenging (Li et al., 2025; Liu et al., 2025; Lv et al., 2025; Wilson and Sletten, 2024; Yang et al., 2025; Ye et al., 2025; Yin et al., 2024; Yuan et al., 2025). A key strategy in designing such probes relies on ring formation/opening mechanisms, where target binding triggers a reversible structural change in the probe’s fluorescent core, altering its optical properties. This design offers high specificity and minimal background signal, but its rational optimization is hampered by insufficient mechanistic understanding. Current research often relies on empirical trial-and-error: while ring-opening/closing is known to modulate fluorescence, the precise link between structural state (closed vs. open), electronic configuration (e.g., electron transfer pathways), and fluorescence output (e.g., quantum yield, emission wavelength) remains unclear. For instance, it is not fully established how ring opening affects intramolecular charge transfer (ICT) efficiency or energy dissipation pathways, which directly govern fluorescence activation.

This knowledge gap significantly increases the cost and inefficiency of probe development, particularly for multifunctional probes integrating targeting, imaging, and therapeutic capabilities. As the demand for NIR probes in precision medicine grows—from early cancer diagnosis to intraoperative navigation—clarifying these mechanisms becomes imperative. Against this backdrop, quantum chemical calculations have emerged as a powerful tool to dissect the photophysical processes of fluorescent molecules at the atomic level, offering insights into light absorption, emission, and electronic structure changes that are difficult to be caught experimentally (Fan et al., 2024; Kanlayakan et al., 2022; Zhao et al., 2024).

By applying quantum chemical calculation methods, this research analyzes key processes including light absorption, emission, electron transfer characteristics, and electronic structure changes of the recently developed NIR fluorescent probes (NIR-pH, NIR-ATP and NIR-Hg) before and after reacting with target analytes (Ren et al., 2021). The goal is to clarify the relationship between the ring structure state (closed vs. open) and fluorescence properties, thereby establishing a theoretical basis for the design and improvement of NIR fluorescent probes targeting specific disease-related analytes.

## Theoretical calculation methods

To systematically investigate the electronic structures, light absorption/emission processes, and fluorescence detection mechanisms of the near-infrared (NIR) fluorescent probes (NIR-pH, NIR-ATP, NIR-Hg) and their corresponding reaction products with target analytes (H^+^, ATP, Hg^2+^), the following theoretical calculation methods were employed.

### Electronic structure calculations

The Gaussian 16 program package was used as the core computational tool, integrating Density Functional Theory (DFT) and Time-Dependent Density Functional Theory (TD-DFT) to calculate the electronic structures of the probes and their sensing adducts (products after reacting with target analytes) (Frisch et al., 2019). Two sets of functional/basis set combinations were selected to address different computational objectives, ensuring accuracy in describing ground and excited state properties: (Deng et al., 2024; Laun and Bredow, 2022; Peng et al., 2023; Su et al., 2013; Yanai et al., 2004):

For optimizing the stable geometric structures and analyzing the electronic characteristics of the probes and their adducts in the ground state (S_0_), the PBE0/def2-TZVPD combination was adopted. This combination is suitable for capturing the basic electronic distribution and bond configurations of molecules in their lowest energy state.

For investigating the stable geometric structures and electronic behaviors in the first excited state (S_1_) (critical for understanding light absorption and emission), the CAM-B3LYP/def2-TZVPD combination with D3 dispersion correction was used. The D3 dispersion correction was introduced to account for weak intermolecular interactions, while the CAM-B3LYP functional effectively describes charge transfer processes, which are key to interpreting the probes’ fluorescence changes. The molecular electronic structures and fluorescence properties in the gas phase and aqueous solution (based on the SMD model) of the fluorescent probes have been calculated and compared, yielding similar results.

These calculations primarily supported three types of analyses:

The electronic transition process from the ground state (S_0_) to the first excited state (S_1_), including the energy of the transition and the nature of electron migration.

The electronic state density (e.g., the contribution of specific molecular regions to the highest occupied molecular orbital (HOMO) and lowest unoccupied molecular orbital (LUMO)), which reveals the distribution of electron density in key orbitals.

The electron transfer distribution during excitation, distinguishing between charge transfer (e.g., from the reaction site region to the benzene ring) and local excitation characteristics.

### Molecular property analysis

To establish the link between molecular structure, electronic behavior, and fluorescence properties of the probes, multiple molecular property analyses were conducted, with key tools and focuses as follows:

π-electron distribution visualization: The Localized Orbital Locator (LOL) of π-electrons were computed to intuitively display changes in π-electron distribution before and after the probe reacted with target analytes (e.g., from interrupted distribution in the closed five-membered ring state to continuous distribution in the open linear chain state).

Spectral and structural parameter analysis: Key parameters related to fluorescence performance were analyzed, including the Stokes shift of the probes, the overlap between absorption and emission spectra, and changes in molecular structure (e.g., differences in structural vibration modes between the ground state and excited state) after reaction with target analytes. These parameters helped explain the mechanism of fluorescence enhancement.

Data processing and visualization: Most of the above analyses (e.g., electron state density calculation, π-electron distribution analysis) were performed using the Multiwfn 3.8 (dev) code, while partial figures (e.g., molecular structure diagrams, electron transfer heatmaps) were generated using VMD 1.9.3 software to present computational results in a clear, visual format (Humphrey et al., 1996; Lu, 2024; Lu and Chen, 2012).

## Results and discussion

**SCHEME 1:**
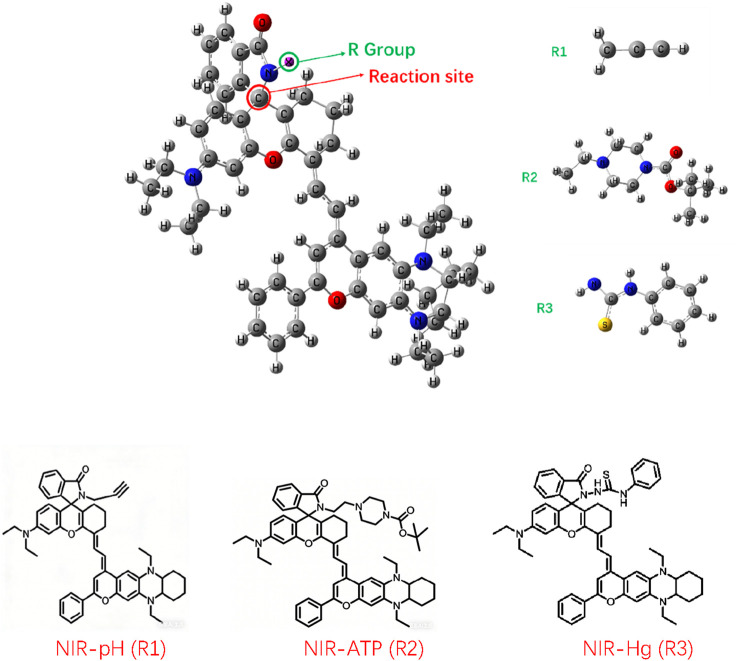
Structure of the infrared fluorescent probe NIR-pH (R1), NIR-ATP (R2) and NIR-Hg (R3).

The basic structure of the infrared fluorescent probe developed by Ren et al. is shown in Scheme 1, with the reaction site for the target analyte located on the C atom connected to N at the junction of the six-membered ring and the five-membered ring in the probe molecule. After the reaction, the five-membered ring is opened to form a linear chain end. Different structures (R) on the chain end give the reaction site different reaction characteristics. The three different chain end structures R in the schematic diagram correspond to three different detection targets: H^+^, ATP, and Hg^2+^and the corresponding probe were labeled by NIR-pH, NIR-ATP and NIR-Hg respectively. When the five-membered ring is closed, all probe molecules exhibit weak fluorescence emission. When the probe molecules react with the target analytes to open the five-membered ring into linear chain ends, the infrared fluorescence of the probe molecules significantly enhances with increasing target analyte concentration, making this series of probes efficient infrared switchable fluorescent probes.

**FIGURE 1:**
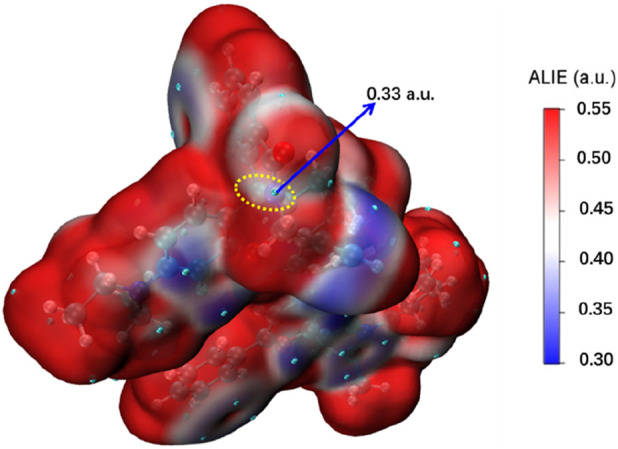
The surface map of ALIE on probe NIR-pH.

The average local ionization energy (ALIE) was a very useful quantity for examining the electrophilic reaction site and activity of a molecule (Han et al., 2025; Politzer et al., 2010). The ALIE value of unsaturated C-N bond in probe NIR-pH (0.33 a.u.) as shown in Figure 1, which could be usually taken as indicator of electrophilic reaction site, indicated the unsaturated C-N bond was the specific recognition site of the probe. After reacting with the H^+^, the change of the molecular and electronic structure and so the obvious variance of the probes’ fluorescent character made the NIR-pH be highly efficient fluorescent probe for detection of acidity.

**FIGURE 2:**
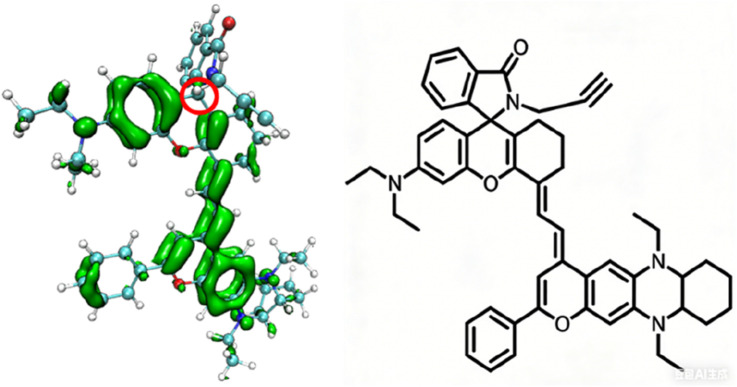
The π-electron density distribution of the optimized NIR-pH probe molecule.

To understand the changes in the electronic structure of the above series of probe molecules before and after reaction with the target analytes, the π-electron density distribution of the optimized NIR-pH probe molecule is shown in Figure 2. Since π electrons are mainly involved in the charge transfer during light absorption and emission processes, only the π-electron part is shown in the schematic diagram.

**FIGURE 3:**
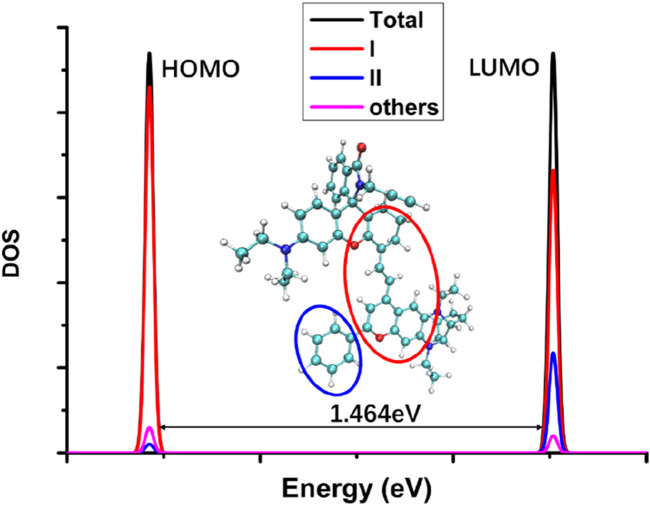
The electronic state density diagram of the probe molecule NIR-pH (DOS: density of electronic states; Total: the whole molecular structure; I: the red circle part; II: the blue circle part; others: the whole molecular structure except the part I and II; the energy gap between HOMO and LUMO was 1.464eV as indicated in the figure).

**FIGURE 4:**
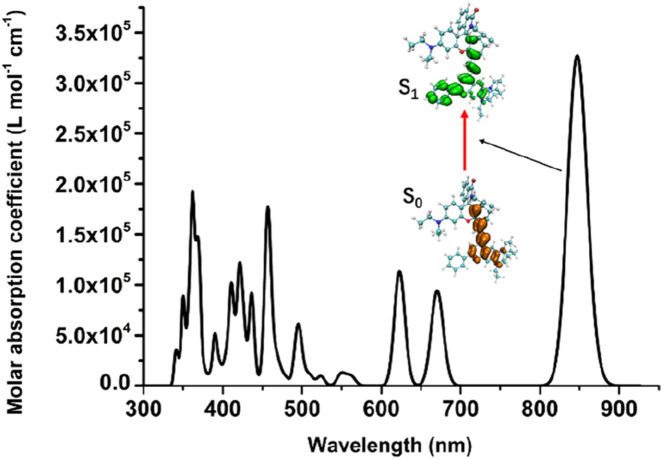
The electron density change associated with its absorption peak from the ground state to the first excited state of NIR-pH.

It can be seen from Figure 2 that when the five-membered ring in the NIR-pH probe molecule is closed, the π-electron distribution in the molecule is interrupted here due to the C-N bonding at the reaction site. During the transition of electrons from the ground state to the excited state, an interruption also occurs at the C-N bond of the reaction site, and some π electrons migrate to the benzene ring part at the other end. This charge transfer characteristic results in low fluorescence intensity of the probe molecule when the five-membered ring is closed. This charge transfer characteristic can be seen from the density diagram of electronic state (DOS) the probe molecule before reaction with the target analyte (Figure 3) and the electron density change associated with its absorption peak from the ground state to the first excited state (Figure 4). Partial DOS (PDOS) represents the curve of contributions of specific fragments to the Total DOS (TDOS). If fragments are properly defined, the nature and main composition of orbitals in different energy ranges can be well grasped through PDOS plots. Obviously, if the union of all defined fragments equals the entire system, the PDOS curves of each fragment will sum up exactly to the TDOS. Based on the charge transfer characteristics during excitation from the S_0_ to S_1_ state, three fragments (part I, part II and part others) in the probe molecule and its detected product were selected to calculate the corresponding PDOS, which were then plotted in the DOS diagram as followed.

**FIGURE 5:**
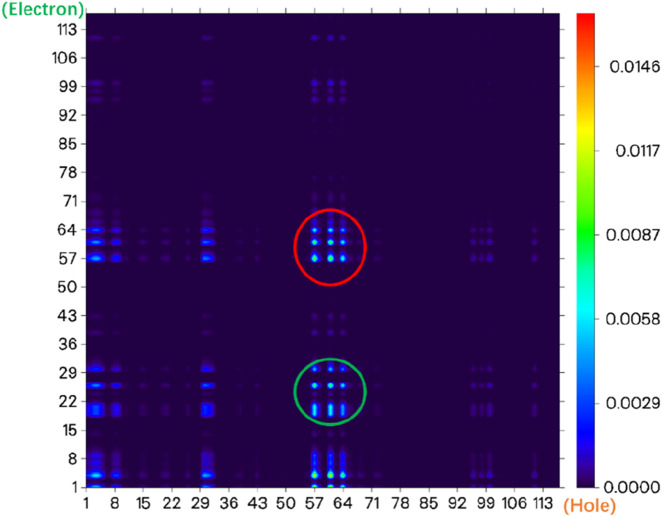
The electron transfer heatmap of the probe molecule NIR-pH excited from the ground state (S_0_) to the first excited state (S_1_).

From the electronic state density (Figure 3), it can be seen that the highest occupied molecular orbital (HOMO) and the lowest unoccupied molecular orbital (LUMO) of the NIR-pH probe molecule mainly come from the contribution of the red elliptical region in the molecular structure. Slightly differently, in addition to the contribution from region I, the LUMO also has a significant contribution from the blue elliptical region II. This reflects that the probe molecule has considerable electron transfer excitation characteristics during the excitation process from the ground state S_0_ to the first excited state S_1_ (corresponding to the transition of electrons from the HOMO to the LUMO). This characteristic can be clearly seen not only from the difference in electron density distribution before and after excitation in Figure 4 but also from the electron transfer heatmap (Figure 5) of the probe molecule excited from the ground state (S_0_) to the first excited state (S_1_). Plotting the transition density matrix between S_0_ and S_1_ states with atomic numbers as the horizontal and vertical coordinates, and using colors to represent the magnitude of transition density values, can conveniently analyze the atomic range involved in electronic transitions from S_0_ to S_1_ as shown in Figures 5, 9. In Figure 5, in addition to the local excitation characteristics shown on the diagonal (red elliptical region), the heatmap distribution in the green elliptical region clearly shows the electron transfer excitation characteristics from the red elliptical region (shown in Figure 3) to the blue elliptical region (benzene ring in Figure 3) during the excitation process.

**FIGURE 6:**
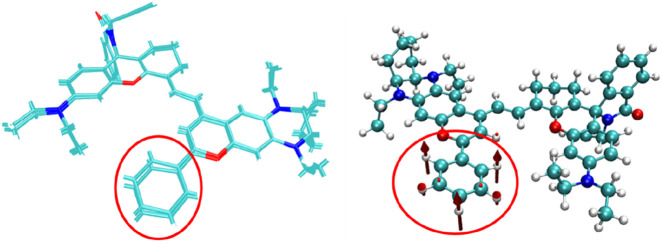
A comparison of the structures of the NIR-pH probe molecule before and after electronic excitation and the out-of-plane vibration of the benzene ring.

A comparison of the structures of the NIR-pH probe molecule before and after electronic excitation (Figure 6) shows that the structural changes between the two mainly occur in the benzene ring region shown in the figure. This is because the closure of the five-membered ring truncates the excitation process of the π electrons on its left part, causing the π electrons to migrate toward the benzene ring instead. This makes the out-of-plane vibration of the benzene ring the main vibration mode for absorbing or radiating energy during the molecular excitation process.

**FIGURE 7:**
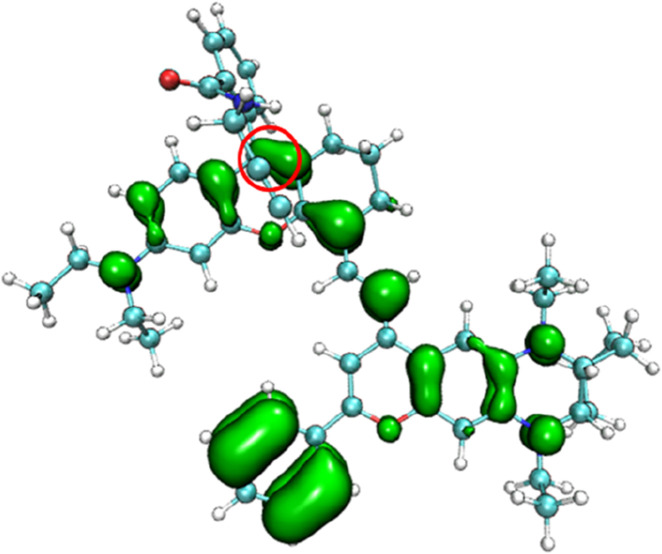
The π-electron density distribution of the product NIR-pH-product.

When the NIR-pH probe molecule reacts with H^+^, the C-N bond in the five-membered ring of the probe serving as the reaction site, cleaves after reacting with the corresponding target analyte. Its five-membered ring is opened into a linear chain end structure. Its π-electron distribution is no longer truncated by the five-membered ring (Figure 7). During the excitation process of the molecule from the ground state (S_0_) to the first excited state (S_1_), no obvious charge transfer characteristic appears; instead, a local excitation characteristic is observed. This change results in a significant enhancement of the infrared fluorescence intensity of the probe molecule after reaction with the target analyte, which becomes the fluorescence detection mechanism of this series of infrared fluorescent probes for various target analytes.

**FIGURE 8:**
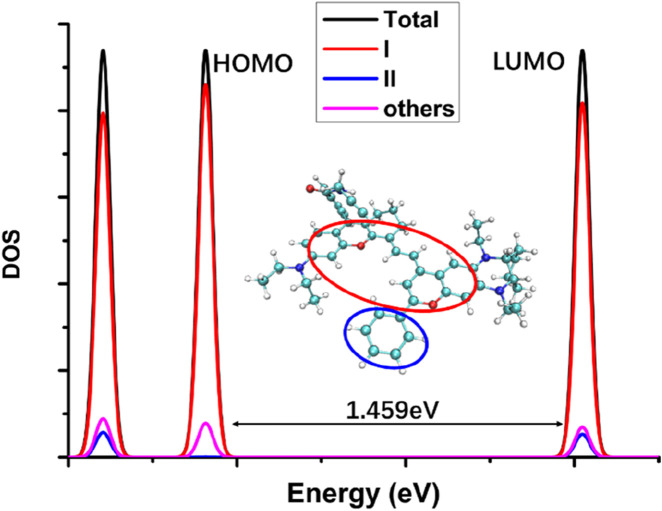
The electronic state density of the NIR-pH-product (DOS: density of electronic states; Total: the whole molecular structure; I: the red circle part; II: the blue circle part; others: the whole molecular structure except the part I and II; the energy gap between HOMO and LUMO was 1.459eV as indicated in the figure).

**FIGURE 9:**
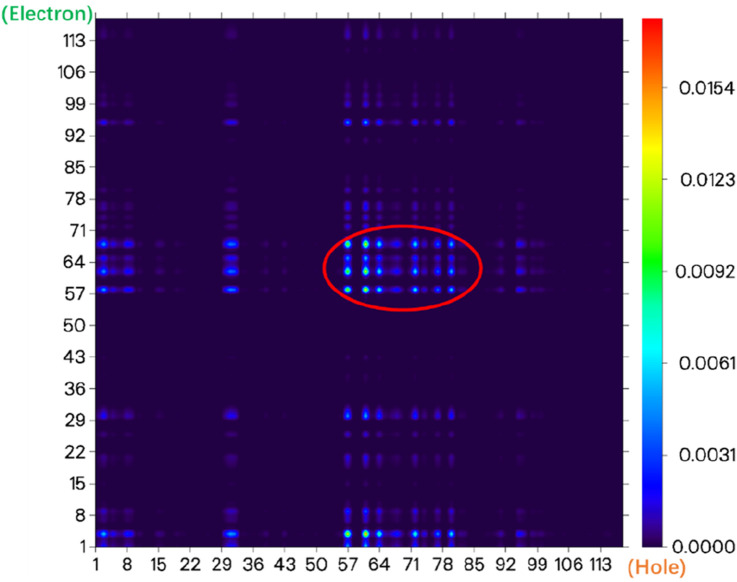
The electron transfer heatmap of the probe molecule NIR-pH-product excited from the ground state (S_0_) to the first excited state (S_1_).

The electronic state density of the NIR-pH-product after reaction with H^+^ (Figure 8) shows that its HOMO and LUMO are mainly contributed by the red elliptical part, with little contribution from other parts of the molecule. This can also be seen from the electron transfer heatmap (Figure 9) of this molecule from the ground state (S_0_) to the first excited state (S_1_). The excitation process associated with the transition of electrons from the HOMO to the LUMO (S_0_→S_1_) shows obvious local excitation characteristics (concentrated in the red elliptical region in Figure 8).

**FIGURE 10:**
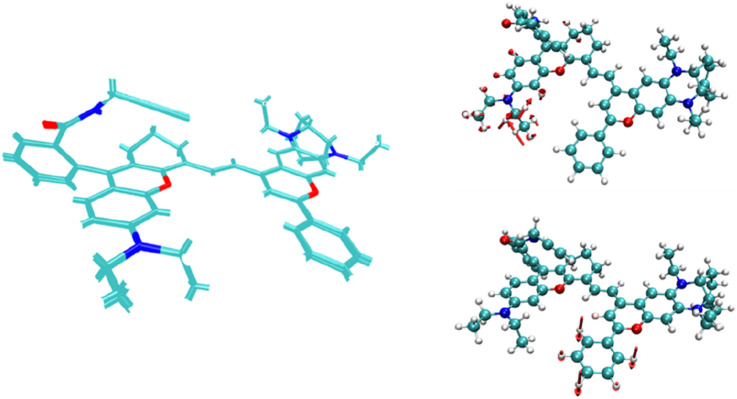
A comparison of the structures of the NIR-pH-product molecule before and after electronic excitation and the corresponding vibration with significant energy contribution to the excitation process.

At the same time, from the structural comparison diagram between the ground state and the first excited state of the probe product after reaction with H^+^ as shown in Figure 10, it can be seen that compared with the NIR-pH probe molecule, the structural changes between the two states are no longer mainly limited to the benzene ring part, and the vibration of the carbon chain part at the other end also makes a significant energy contribution to the excitation process. This phenomenon reflects that after the opening of the five-membered ring, the π electrons involved in the S_0_→S_1_ excitation process have a wider range of expansion in the product, which also leads to the enhancement of the infrared fluorescence intensity of the product. The infrared fluorescence of the series probes NIR-ATP and NIR-Hg also has similar detection mechanisms, and their relevant calculation results are provided in the supporting information for reference.

The detail analysis of optical excitation and emission process within all three stable probe conformations and the corresponding products were summarized in Tables 1, 2. It could be seen that the calculated central wavelength of excitation and emission process were consistent with the experimental value reported in reference (Ren et al., 2021). As can be seen from the Tables 1, 2, the electronic transitions associated with the fluorescence emission of the studied near-infrared fluorescent probes and their corresponding detected products mainly occur between their HOMO and LUMO. Compared with the fluorescent probes, the vibrational intensities of the detected products were significantly enhanced, and their absorption and emission wavelengths exhibited slight red shifts. These calculation results were consistent with the experimental phenomena, which not only provides a theoretical explanation for the experimental observations but also verifies the rationality of the selected theoretical methods in this study for investigating the relevant fluorescence mechanisms.

**TABLE 1:** The main electron excitation processes in the probe molecule.

Probe and Product	Electronic transition^a^	Excitation energy	Oscillator strengh	Composition^b^	CI^c^
NIR-pH	S_0_→S_1_	847 nm	1.0428	H→L	0.7514
NIR-pH-product	S_0_→S_1_	850 nm	1.1084	H→L	0.8064
NIR-ATP	S_0_→S_1_	837 nm	0.9907	H→L	0.8046
NIR-ATP-product	S_0_→S_1_	845 nm	1.0248	H→L	0.8067
NIR-Hg	S_0_→S_1_	840 nm	1.4518	H→L	0.7049
NIR-Hg-product	S_0_→S_1_	849 nm	1.2109	H→L	0.7045

^a^ Only the excited states with oscillator strength larger than 0.1 were considered.

^b^ H stands for HOMO, and L stands for LUMO.

^c^ Coefficient of the wave function for each excitation was in absolute value.

**TABLE 2:** The main emission processes in the probe molecule.

Probe and Product	Electronic transition^a^	Emission energy	Oscillator strengh	Composition^b^	CI^c^
NIR-pH	S_1_→S_0_	906 nm	0.0048	H→L	0.6914
NIR-pH-product	S_1_→S_0_	912 nm	0.9978	H→L	0.6873
NIR-ATP	S_1_→S_0_	901 nm	0.0026	H→L	0.7146
NIR-ATP-product	S_1_→S_0_	909 nm	1.0235	H→L	0.7048
NIR-Hg	S_1_→S_0_	918 nm	0.0023	H→L	0.7053
NIR-Hg-product	S_1_→S_0_	925 nm	1.0304	H→L	0.6927

a,b,c same indication as in Table 1.

## Conclusion

This study systematically clarifies the fluorescence detection mechanism of NIR fluorescent probes (NIR-pH, NIR-ATP, NIR-Hg) based on five-membered ring opening/closing through quantum chemical calculations, yielding three key conclusions:

Average Local Ionization Energy (ALIE) calculations identified the unsaturated C-N bond as the specific electrophilic reaction site (e.g., ALIE = 0.33 a.u. for NIR-pH), confirming its role in analyte binding and ring opening. The ring structure state determines fluorescence intensity: In the closed five-membered ring state, the C-N bond at the reaction site interrupts π-electron distribution. This causes electron transfer during excitation, restricts π-electron activity to the benzene ring region, and results in weak fluorescence of the probes. When the ring opens after reacting with target analytes, π-electron truncation is eliminated, leading to a significant enhancement in fluorescence intensity.

Excitation mode shift drives fluorescence enhancement: The closed ring state induces charge transfer excitation (from the reaction site region to the benzene ring), while the open ring state switches to local excitation. Additionally, the open linear chain structure expands the range of π-electrons involved in the S_0_→S_1_ excitation process (incorporating both benzene ring and carbon chain regions), further boosting fluorescence.

Calculation results validate mechanism reliability: The calculated excitation and emission central wavelengths of all three probes and their reaction products are consistent with previously reported experimental data. This confirms the accuracy of the proposed detection mechanism and provides a robust theoretical framework for future probe development.

In summary, this study establishes a clear link between the ring opening/closing behavior, electronic structure changes, and fluorescence properties of NIR probes. The insights gained can guide the rational design of high-performance NIR fluorescent probes targeting a broader range of disease-related analytes, promoting their application in biological imaging and medical diagnostics.

## Data Availability

The original contributions presented in the study are included in the article/Supplementary Material, further inquiries can be directed to the corresponding authors.
