# Supplementary material for: Investigation on the fluorescence detection mechanism of NIR fluorescent probes based on intramolecular spiro cyclization
Source: Front Chem. 2026 Jan 8;13:1756681. doi: 10.3389/fchem.2025.1756681 (PMC12823956; doi:10.3389/fchem.2025.1756681)
Supplement: Supplementary file 1 [file DataSheet1.docx]

**Investigation on the Fluorescence Detection Mechanism of NIR Fluorescent Probes Based on intramolecular spiro cyclization**


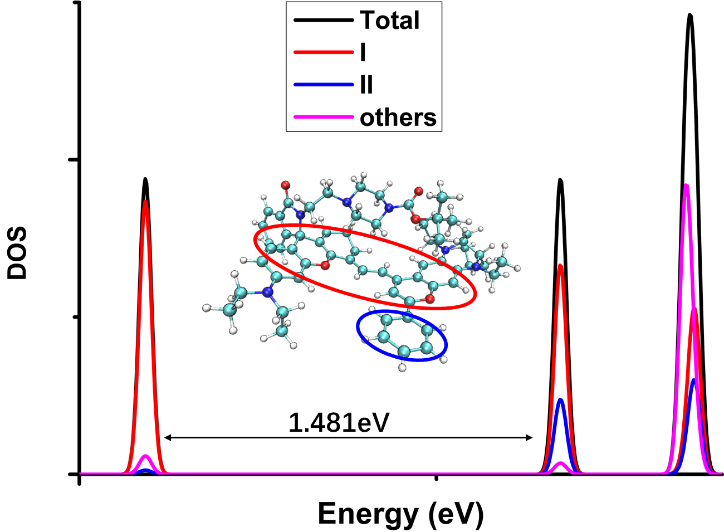


Figure S1 The electronic state density diagram of the probe molecule NIR-ATP


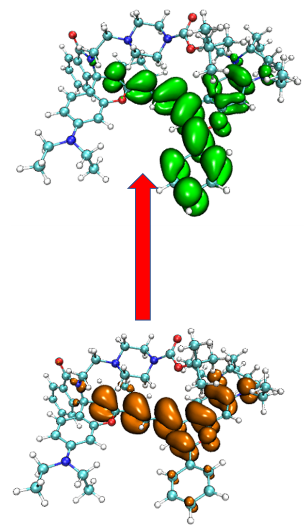

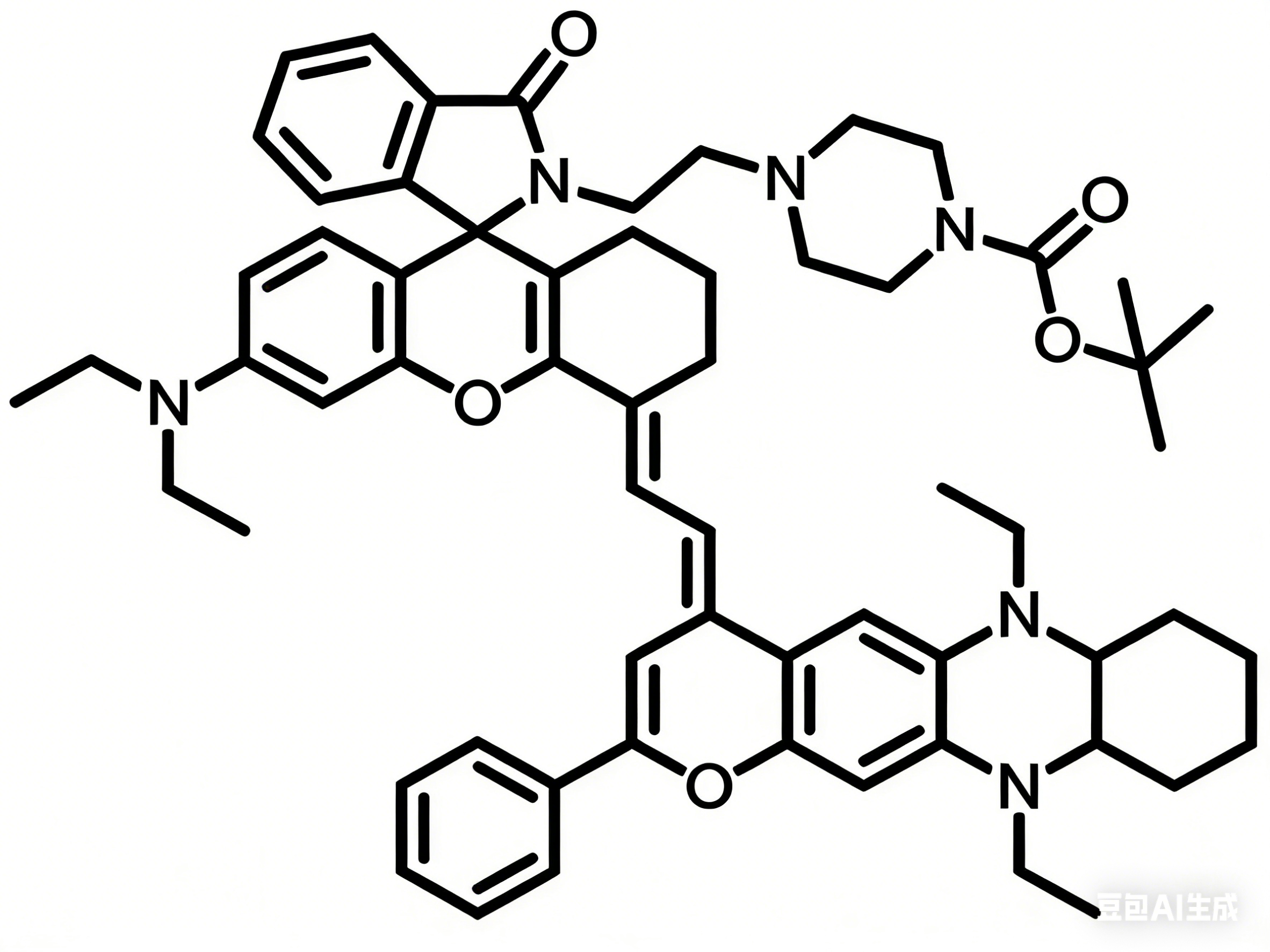


Figure S2 The electron density change from the ground state to the first excited state of NIR-ATP


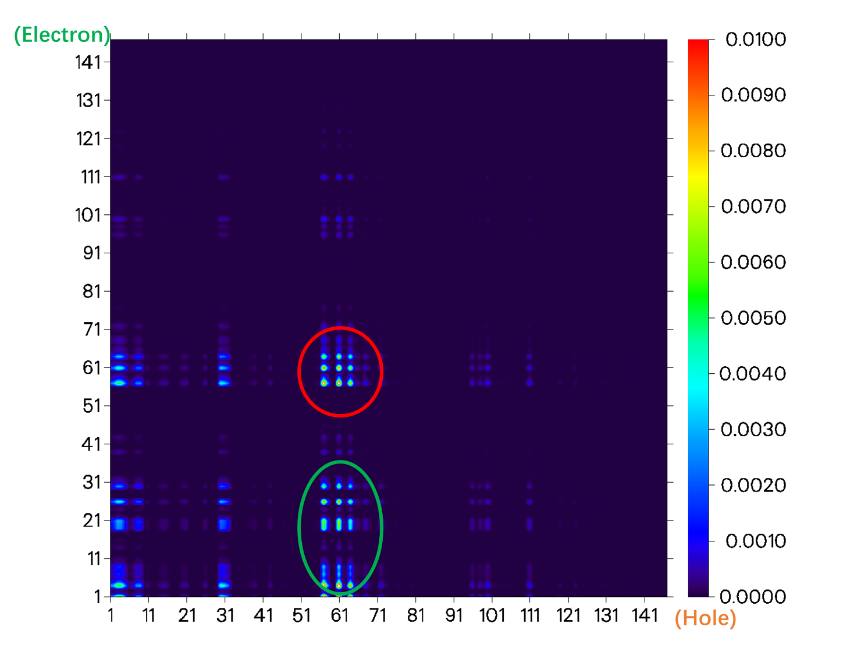


Figure S3 The electron transfer heatmap of the probe molecule NIR-ATP excited from the ground state (S₀) to the first excited state (S₁)


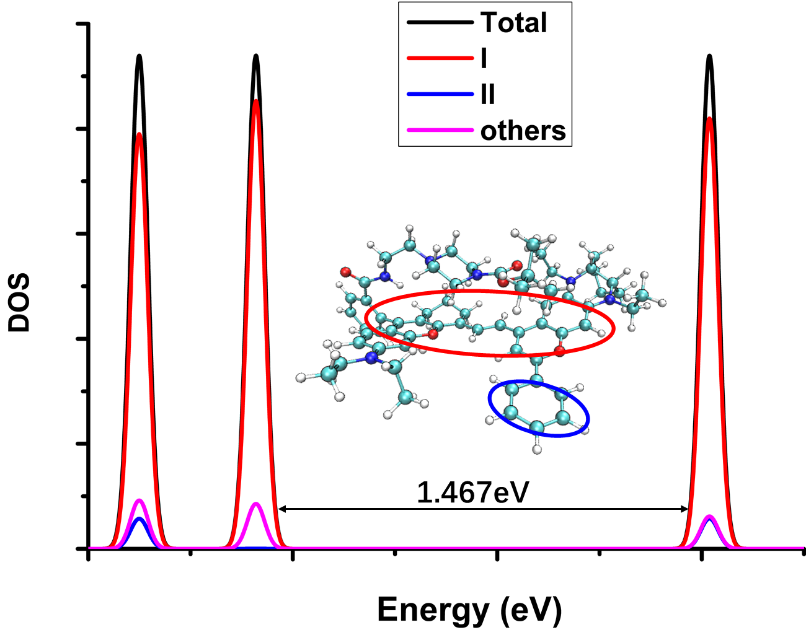


Figure S4 The electronic state density of the NIR-ATP-product


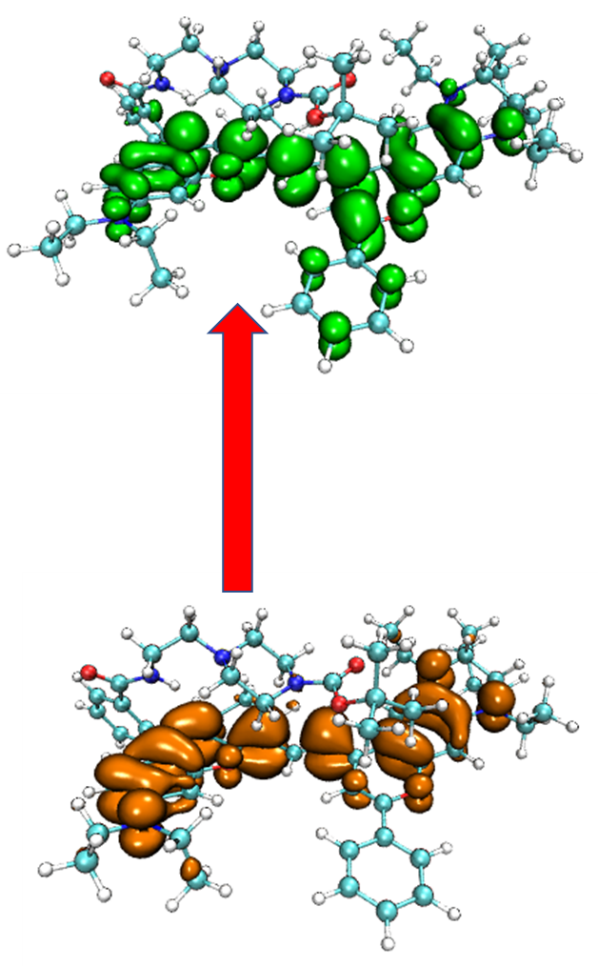


Figure S5 The electron density change from the ground state to the first excited state of NIR-ATP-product


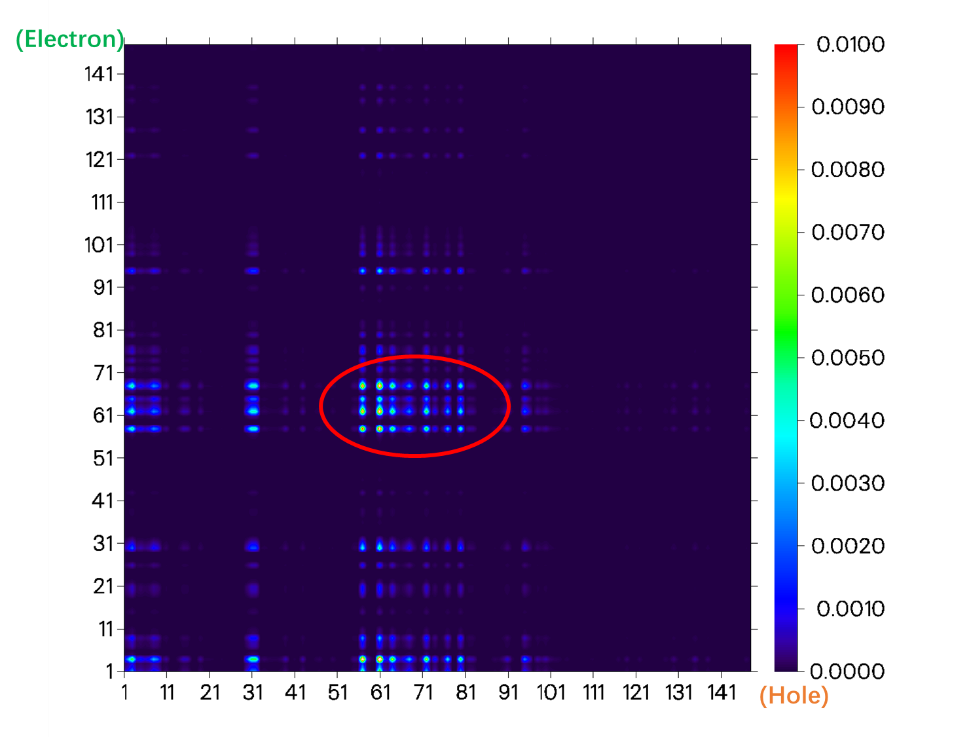


Figure S6 The electron transfer heatmap of the probe molecule NIR-ATP-product excited from the ground state (S₀) to the first excited state (S₁)


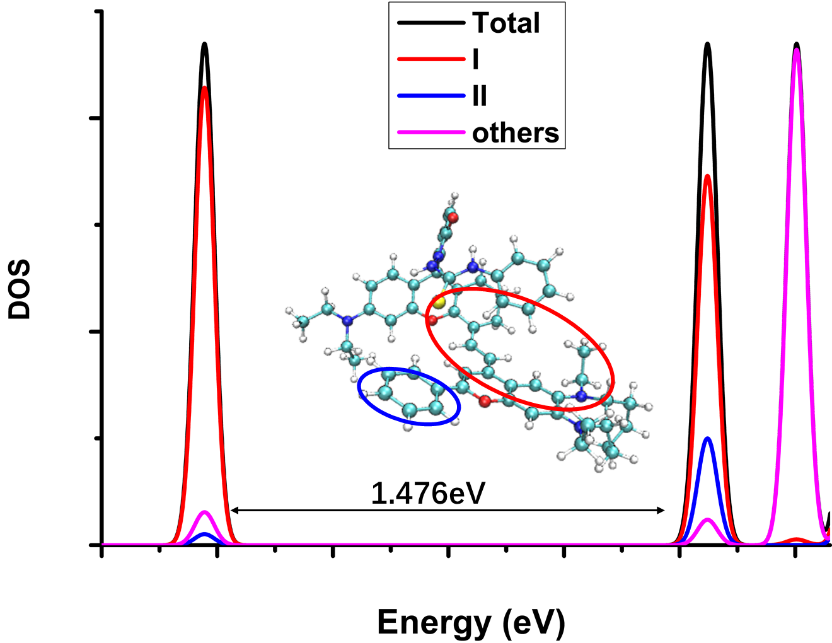


Figure S7 The electronic state density of the NIR-Hg


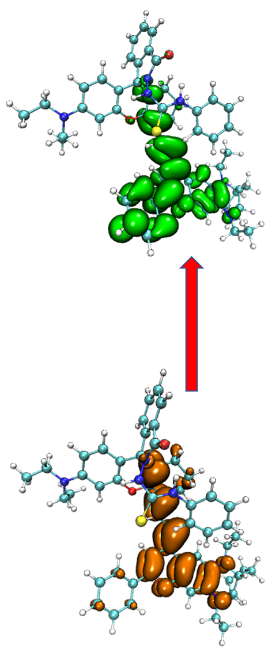

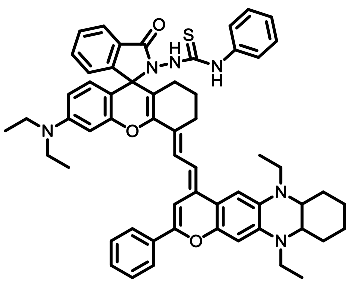


Figure S8 The electron density change from the ground state to the first excited state of NIR-Hg


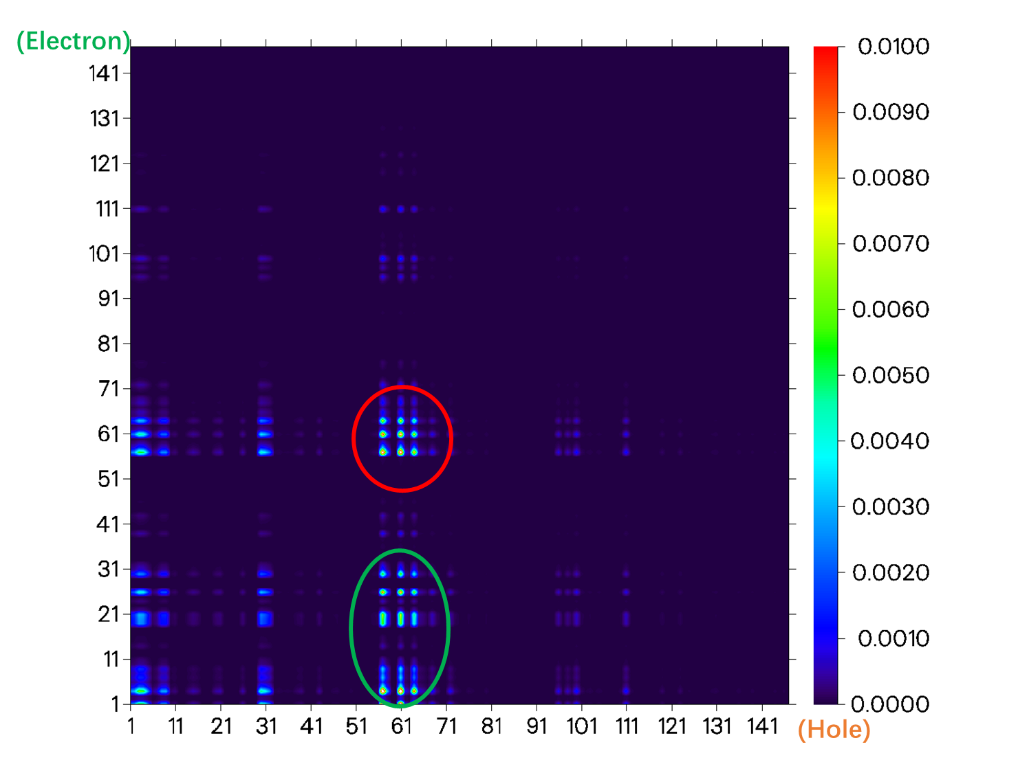


Figure S9 The electron transfer heatmap of the probe molecule NIR-Hg excited from the ground state (S₀) to the first excited state (S₁)


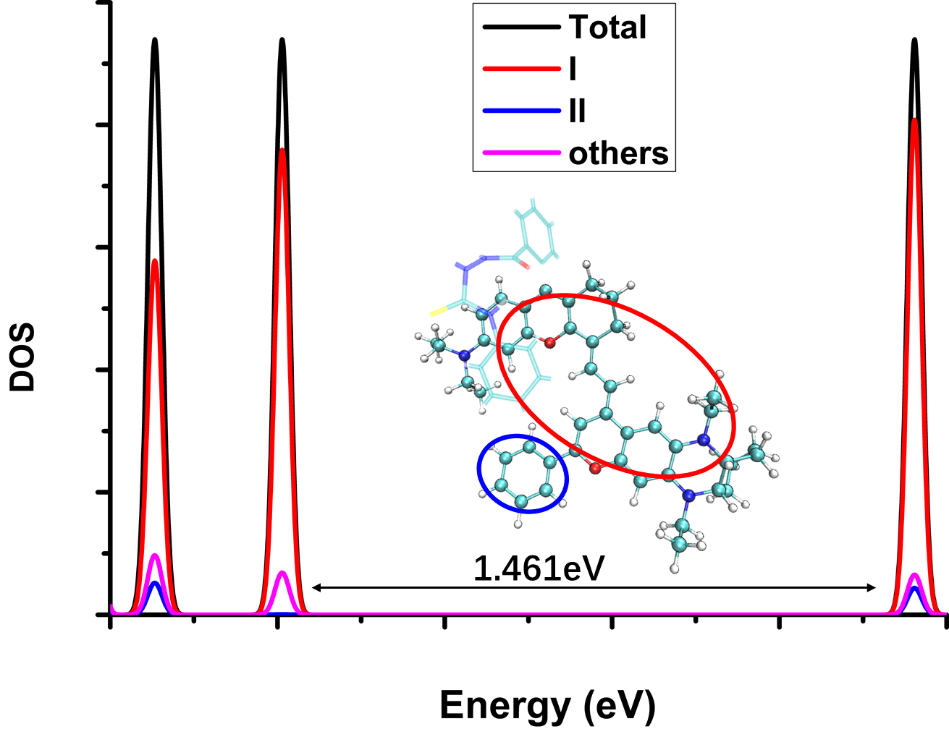


Figure S10 The electronic state density of the NIR-Hg-product


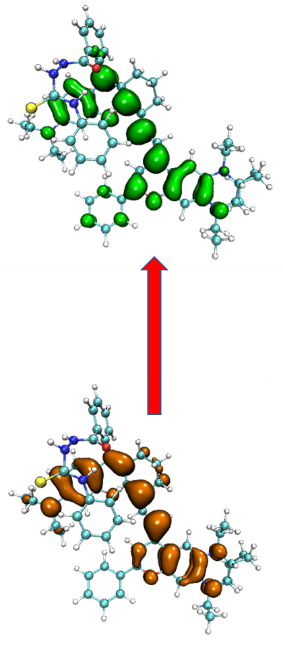


Figure S11 The electron density changes from the ground state to the first excited state of NIR-Hg-product


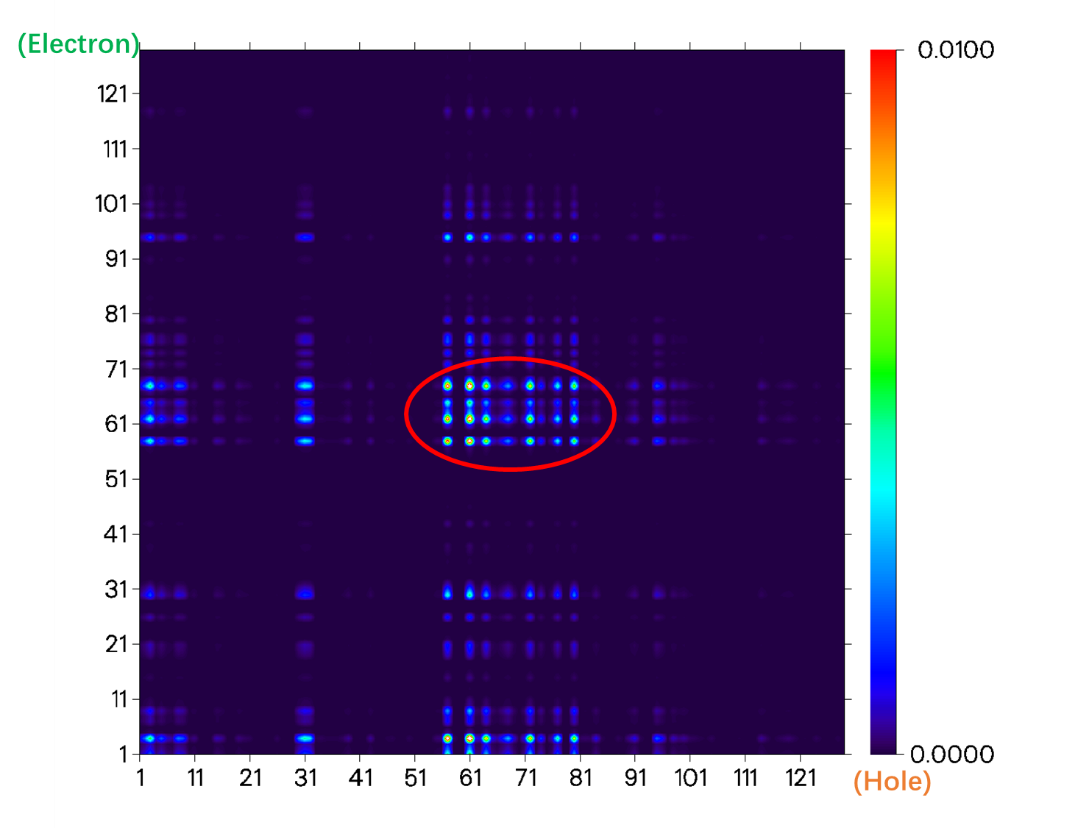


Figure S12 The electron transfer heatmap of the probe molecule NIR-Hg-product excited from the ground state (S₀) to the first excited state (S₁)
